# A scoping review and modelling of predictors of an abnormal Thompson score in term neonates in low-resource settings

**DOI:** 10.1038/s41598-025-96566-7

**Published:** 2025-04-10

**Authors:** Khan Nushrat, Mugwagwa Edna, Cortina-Borja Mario, Catherall Ellie, Fitzgerald Felicity, Chimhuya Simbarashe, Chimhini Gwendoline, Gannon Hannah, Crehan Caroline, Mangiza Marcia, Heys Michelle

**Affiliations:** 1 Department of Primary Care and Public Health, Faculty of Medicine, Imperial College London, London, UK; 2 London School of Hygiene and Tropical Medicine, London, UK; 3 Population, Policy and Practice Research and Teaching Department, Great Ormond Street Institute of Child Health, University College London, London, UK; 4 Department of International Development, London School of Economics and Political Science, London, UK; 5 Department of Infectious Disease, Imperial College London, London, UK; 6 Department of Paediatrics and Child Health, University of Zimbabwe, Harare, Zimbabwe; 7 Sally Mugabe Central Hospital, Harare, Zimbabwe

**Keywords:** Newborn care, Neonatal encephalopathy, Predictive modeling, LMIC, Low-resource settings, Health care, Medical research, Risk factors, Signs and symptoms

## Abstract

Clinical risk scores, such as Thompson score, are useful alternatives to identify neonatal encephalopathy in low-resource settings where adequate training and equipment are often unavailable. An understanding of the clinical predictors of abnormally high Thompson score values would be beneficial to identify term neonates with suspected neonatal encephalopathy. A scoping review was conducted to identify a set of a priori neonatal and maternal variables associated with neonatal encephalopathy. Next, a prospective study of all term neonates admitted to Sally Mugabe Central Hospital in Zimbabwe between October 2020 and December 2022 was conducted to develop a predictive statistical model of abnormal (> 10) Thompson score. In total 45 articles were identified from searching Medline, Scopus and Web of Science and 10 articles were selected. Five studies were conducted in countries in Asia and five in Africa. Of 6,054 neonates who met the inclusion criteria, 4.06% (*n* = 246) had an abnormal Thompson score at admission with a case fatality rate of 589 per 1000 admissions. Among these neonates, 90.65% (*n* = 223) had a low Apgar score (*p* < 0.001). 40 candidate predictors were identified, of which 20 predictors were selected as the most important. Six risk factors were predictive of neonates at risk of abnormal Thompson score, including low neonatal heart rate (aOR = 0.97), temperature lower than 36.5 °C (aOR = 2.24), head swelling (aOR = 2.19), other maternal risk factors of sepsis excluding offensive liquor and premature rupture of membranes (aOR = 1.97), meconium-stained umbilicus (aOR = 1.79), and not crying at birth (aOR = 2.58). These identified risk factors should be prioritised before conducting a Thompson score assessment in resource-poor settings, and local clinical guidelines should incorporate these into the clinical management of at-risk neonates.

## Introduction

Progress has been made globally in reducing under- 5 mortality; however, mortality in newborns (below 28 days of age) remains disproportionately high, accounting for nearly half of under- 5 deaths^1^. Of the 2.4 million newborn deaths globally, 90% occur in low- and middle-income countries, with the highest neonatal mortality rates occurring in sub-Saharan Africa^2^. Infection, prematurity, and neonatal encephalopathy (NE) are the three most common causes of newborn mortality^3^. In addition to causing an estimated one million deaths worldwide every year, NE is a cause of significant life-long disability^4^.

NE is an abnormal neurological function in the first few days of life in a baby born at term (gestational age of 37 weeks or more), which can be characterised by depressed levels of consciousness, seizures, abnormal tone, respiratory depression and impaired feeding^5^. This can be caused by perinatal asphyxia, metabolic disorders, perinatal infections and placenta abnormalities^6^. Another neonatal condition called Hypoxic Ischaemic Encephalopathy (HIE) is also characterised as NE in the presence of perinatal asphyxia^7^. In high-resource settings, the diagnosis of NE and identification of its underlying risk factors are determined by clinical assessment and investigation of the mother and baby including cord blood arterial blood gases, neuroimaging procedures such as continuous amplified electroencephalogram (EEG) and magnetic resonance imaging (MRI)^8^. However, clinical diagnosis and management of NE can be difficult in low-resource settings due to lack of competent, skilled care providers and provision of basic and emergency obstetric care^9^. Continuous amplified EEG and MRI are rarely available in such settings where diagnosis is made on clinical features alone, making it often challenging to diagnose NE with certainty.

Clinical prediction scores combining clinical features with EEG result have been developed to categorise severity of NE and predict neurodevelopmental sequelae in neonates with a well-defined episode of foetal distress, such as the Sarnat and Sarnat score^10^. However, this score has two key limitations, namely the reliance on EEG and a need to incorporate a well-defined episode of foetal distress—neither of which are routinely available in low-resource settings due to lack of monitoring during labour. To address these gaps, Thompson et al.^11^developed a simplified score incorporating findings on clinical examination alone. It relies on nine clinical features and assigns a score of 0 to 2 or 3 per feature to characterise peak severity of encephalopathy in term neonates (range 0–22). Infants with a score of 0 are considered normal, those with a score between 1 and 10 are considered to have a diagnosis of mild NE,a score of 11–14 is considered indicative of moderate NE and a score of 15–22 as severe NE. While a high value of Thompson score within the first seven days of life is a sensitive predictor of abnormal neurodevelopmental outcome^12–14^the criteria for measuring an infant’s Thomspon score are unclear and difficult to operationalise in neonatal units where clinical staff may not be sufficiently skilled in identifying clinical signs of NE^11^. Therefore, the underlying reasons for suspecting NE and subsequently wanting to measure the Thompson score are neither clearly stated nor uniform across different studies^15^^,^^16^. Nevertheless, these studies provide valuable evidence for the various maternal and neonatal factors that may trigger clinicians to use the Thompson score. Therefore, further work is needed to clarify the prognostic value of the Thompson score in the first few hours of life or at admission to a neonatal unit in order tohelp the healthcare providers identify the neonates at risk of NE and prioritise for assessment.

One such approach to identify at-risk neonates is to quantify risk factors that contribute to abnormal Thompson score using routine data. While individual level routine data can be scarce in low-resource settings, Neotree, a digital intervention that has been developed to improve neonatal care and outcomes can be a valuable resource (^17,18–20^. Neotree provides data capture, clinical decision support and education in newborn care in low-resource settings and it has been embedded as part of daily care at two hospitals in Zimbabwe (Sally Mugabe Central Hospital and Chinhoyi Provincial hospital) and one hospital in Malawi (Kamuzu Central Hospital)^20^. A clinical diagnostic algorithm for NE is being developed within this wider programme of work where the workstreams included digitalisation of international and national evidence-based guidelines describing risk factors for NE, and a Delphi review of the proposed NE algorithm by the Neotree team by a panel of international experts in newborn care. However, the experts concluded that there was a lack of evidence for these risk factors to be used for NE diagnosis and recommended the use of validated encephalopathy scores such as the Thompson score instead^21^. However, due to a shortage of skilled healthcare professionals in low-resource settings, it is not always feasible to conduct a Thompson score assessment on all term neonates admitted to newborn care units^18^. Furthermore, it remains unclear which neonates should be prioritised for a detailed neurological and Thompson score assessments^22^.

The goal of this study is to characterise neonates born in low-resource settings who are at risk of NE, where routine access to blood tests or neurological investigations can be scarce. Our aim is to conduct a scoping review to determine a priori predictors of abnormal Thompson score. Then we subsequently develop a statistical predictive model for abnormal Thompson score values using routine data that can be incorporated into clinical pathways for newborn care to enable healthcare providers to consistently and explicitly decide which infants to screen for NE. This would help us understand the role of neonatal and maternal predictors which identify neonates in need of measuring their Thompson score.

## Methods

### Scoping review

To identify maternal and neonatal clinical and demographic factors that might be associated with abnormal Thompson score and thus with a high likelihood of diagnosis of NE, three search engines were used to find articles published up to 31 July 2022: Medline (PubMed), Scopus and Web of Science. These searches were conducted between 02–14 August 2022. The PCC (Population, Concept, Context) terms below were used for the literature search with the following primary research question: Which maternal, perinatal and neonatal factors are predictive of NE cases with abnormal Thompson score among neonates born in low-resource settings? The exact search strings are included in the supplementary material (Appendix A):

- Population: Neonates born in low resource settings (due to limited studies conducted in low-resource settings and variations in gestation age range used, a specific gestational age threshold was not used).
- Context: NE cases with abnormal Thompson score, Sarnat score or Hypoxic ischaemic encephalopathy score.
- Context: Low-resource settings or low- and middle-income countries.

Titles and abstracts were evaluated for eligibility by two reviewers (EC and NK). Inclusion criteria for full-texts: a) neonates; b) with a diagnosis of (i) hypoxic ischaemic encephalopathy or NE or (ii) birth asphyxia or (iii) perinatal asphyxia; c) assessed using Thompson score, Sarnat score or Hypoxic ischaemic encephalopathy score; d) studies that also included maternal factors; and e) studies in low-income, low- and middle-income countries or low-resource settings. Exclusion criteria: a) animal studies; b) studies not published in English; c) use of scoring systems other than Thompson score, Sarnat score or Hypoxic ischaemic encephalopathy score; d) clinical trials; e) not including risk factors or predictors of NE. Data extraction included the following information: study design, sample size, study setting, scoring system used in the study and inclusion criteria for neonates in the study.

## Modelling of risk factors associated with abnormal Thompson score

### Study population and setting

All neonates born at term (gestation age ≥ 37 weeks) who were admitted in the neonatal unit of Sally Mugabe Central Hospital in Zimbabwe and were assessed for Thompson score were included in the study. The hospital has a 100-bed neonatal unit and on average 225 monthly admissions. Available equipment includes oxygen and non-invasive ventilation but there is no access to brain imaging, EEG, or cord blood samples to clinically assess NE in neonates^23^. 1^st^ October 2020 to 31^st^ December 2022 was selected as the study period as Thompson scores were regularly taken for all term neonates during this time.

This is a sub-study of the wider Neotree project which has research ethics approval from the Harare Central Hospital Research Ethics Committee (Reference number HCHEC070618/58) and UCL Research Ethics Committee (Reference number 5019/004), including the analysis carried out in this study. This sub-study is registered with the UCL Great Ormond Street Institute of Child Health Research and Development (R&D) Office (R&D number 20PP42). Routine data used for this research were collected at the point of admission and discharge of neonates at the hospital using Neotree and therefore, the need for consent from individuals was waived by the aforementioned ethics committees. Only pseudonymised, de-identified data were made available for research purposes. All methods were performed in accordance with relevant guidelines and regulations.

### Main outcome measure

Thompson et al.^11^ showed that in normothermic infants, a score > 10 in the first seven days of life can predict abnormal neurodevelopmental outcomes with 100% sensitivity and 61% specificity. Therefore, the main outcome is Thompson score > 10 at admission.

### Predictive variables

The predictive variables were derived from the scoping review in step 1 and from existing national and international guidelines that were explored in a previous Delphi study^21^ (Appendix B). Neotree data dictionary was reviewed to identify which predictors are included in the data collected through Neotree and the final set of predictive variables was created.

### Data analysis

Missing data in categorical variables were coded as a ‘missing’ category. Numeric predictive variables were assessed to ascertain whether data were missing at random or not at random and imputed using conditional medians. Generalised linear modelling was used to determine the effect of selected a priori variables on the probability that a neonate had a Thompson score > 10. Association between categorical variables was analysed using the χ^2^ test. To prevent overfitting, the dataset was limited to only those input variables that were most predictive for the outcome variable. Logistic regression models were fitted for the final set of variables and Akaike’s Information Criterion (AIC) was used to compare goodness-of-fit.

All analyses were performed in the R statistical language (version 4.1.2) on R Studio^24^.

## Results

### Scoping review

**Fig. 1:**
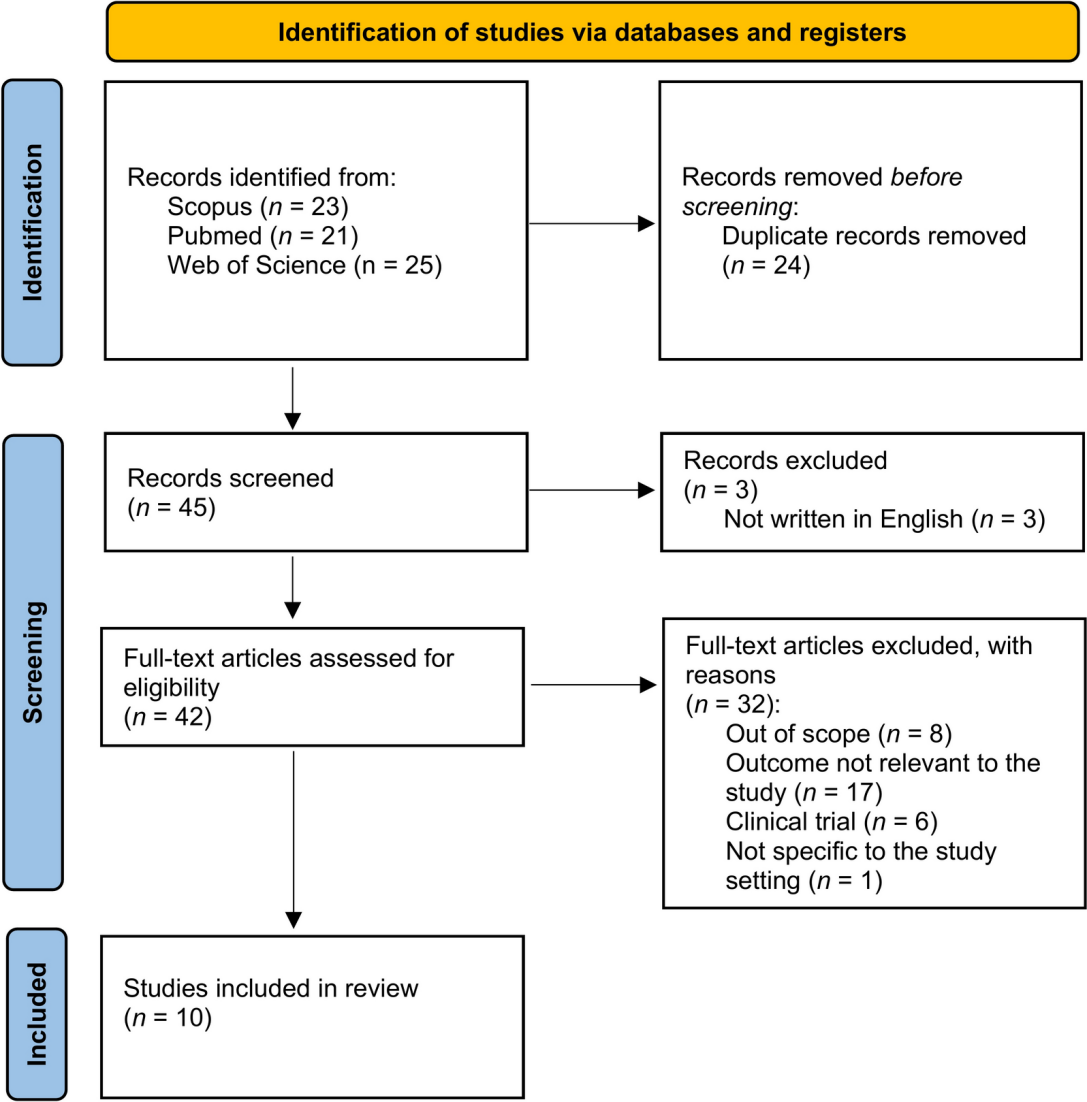
PRISMA flow diagram.

In total 45 papers were identified from three databases and three were excluded for not meeting the language criteria. 42 articles were assessed for eligibility and 32 were excluded for reasons included in Fig. 1. This resulted in 10 papers that described Thompson score or Sarnat grading or HIE score in neonates in low-and-middle income countries with known or suspected diagnosis of NE (or HIE/BA/PA). These papers included descriptions of maternal, perinatal or neonatal factors that might be associated with these diagnoses.

Table 1includes the details of the studies selected, including study type, sample size, study setting, inclusion criteria, and the scoring system used. Out of 10 identified studies, three studies were prospective cross-sectional^25–27^^,^two were prospective observational^16,28^^,^ two were prospective cohort studies^29,30^, and three were retrospective^31^^,^Shah et al., 2005,^32^. These studies were conducted in India (*n* = 3), Nepal (*n* = 1), Thailand (*n* = 1), Democratic Republic of Congo (*n* = 2), South Africa (*n* = 2), and Tanzania (*n* = 1). Sample sizes ranged from 20 to 145. All studies described term neonates admitted with a diagnosis of perinatal asphyxia, with the exception of Sadhanidar et al.^27^ who assessed both term and pre-term neonates who sustained perinatal asphyxia. Low Apgar score (< 6 or ≤ 7) was included as an inclusion criterion to determine perinatal asphyxia in seven out of 10 of the studies and six studies described the use of Thompson score to determine NE.

**Table 1:** Studies selected from the scoping review.

Study	Sample	Study design	Setting	Inclusion criteria	Scoring system used
Bhagwani et al.^25^	145	Prospective, cross-sectional	Hindu Rao Hospital, New Delhi, India	Full-term neonates (gestation age not defined) with low-Apgar score, i.e., 1 min score of ≤ 7	Thompson score
Biselele et al.^26^	50	Prospective, cross-sectional	University Hospital of Kinshasa, Congo	Term and near-term newborn (≥ 36 weeks)	Sarnat grading and Thompson score
Biselele et al.^16^	20	Prospective, observational	University Hospital of Kinshasa, Congo	Term and near-term newborn (≥ 36 weeks), Apgar < 6 after 5 min, Resuscitation until 10 min after birth, pH < 7 and base excess more than 16 on umbilical cord blood or any blood sample (arterial, venous or capillary) within 60 min of birth, Thompson score > 5 one hour after birth	Thompson score
Doreswamy and Ramakrishnegowda^28^	55	Prospective observational	India	Neonates > 35 weeks of gestation with birth weight ≥ 1800 g and requiring resuscitation at birth	Thompson score
Futrakul et al.^31^	84 newborns with perinatal asphyxia	Retrospective	King Chulalongkorn memorial hospital, Thailand	Term neonates (≥ 36) weeks of gestation with Apgar score < 6 at 1 min	Modified Sarnat-Sarnat Score
Horn et al.^29^	41	Prospective cohort study	Three hospitals in cape Town, South Africa	Gestation week ≥ 36 and birth weight ≥ 2000 g, with signs of encephalopathy after age 10 min but before age 5 h. 10-min Apgar score < 7 or continued respiratory support at age 10 min	Thompson score
Mwakyusa et al.^30^	140	Prospective cohort study	Muhimbili national hospital, Dar-es-Salaam, Tanzania	Full-term neonates with low-Apgar score (i.e. a 5 min score ≤ 7) or post-asphyxial symptoms admitted within 24 h of delivery or with symptoms and signs of HIE	HIE score (modified Sarnat scoring system)
Sadhanidar et al.^27^	50	Prospective cross-sectional	A tertiary care government hospital in Guwahati, Assam, India	Both preterm and term neonates sustaining perinatal asphyxia with clinical diagnosis of HII were included to compare MRI findings and clinical staging of Sarnat score	Sarnat and Sarnat Staging system
Singh et al.^33^	50	Retrospective	B.P. Koirala Institute of health sciences, dharan, Nepal	Gestational age ≥ 37 weeks with Apgar score ≤ 6 at 5 min	Sarnat and Sarnat Staging system
Stofberg et al.^32^	29	Retrospective	A district hospital in Cape Town, South Africa	Gestational age ≥ 36 weeks, birth weight ≥ 1.8 kg, a sentinel event in the perinatal course, Apgar score ≤ 7 at 5 min, prolonged resuscitation needed at birth (more than 10 min), proven acidosis within the first hour or life, and other clinical features	aEEG and Thompson score

In a study to understand the clinical profile of asphyxiated newborns, Shah et al. (2005) found that common presentations of HIE had reflexes, seizures, lethargy, and pupillary abnormalities. Subtle seizure, meconium-stained liquor, post-term, and lack of antenatal check-up were reported as key risk factors by Bhagwani et al.^25^. Similar findings were also reported by Futrakul et al.^31^. The authors retrospectively modelled the risk factors of HIE and found inappropriate antenatal care, post-term gestation, vacuum extraction, male gender, prolapsed cord, and Apgar scores at 1 and 5-min to be significant risk factors of HIE. High maternal age (median 31 years) and a high incidence of pre-eclampsia were found to be associated in the study sample of Biselele et al.^26^. The authors compared Thompson score and Sarnat score and found significant correlation between the two scores, as well as with mortality and individual scoring system. Sadhanidar et al.^27^ compared MRI findings with clinical staging of Sarnat score and Apgar score in both term and pre-term neonates. The study suggested that Apgar score 7 or less more commonly affected both groups and found more severe cases of brain injuries in pre-term neonates.

Biselele et al.^16^ studied the evolution of Thompson score over the first 6 h of life and found that the scores change over this time. In contrast, Mwakyusa et al.^30^ reported developmental outcome at 6 months of age of 140 neonates diagnosed with birth asphyxia and found that higher Thompson scores were associated with increased risk of death, seizures and poor neurological outcome. Horn et al.^29^ found that Thompson score could be used to predict death or a persistently and severely abnormal amplitude-integrated electroencephalogram (aEEG) at 48-h. The authors suggested that a score ≥ 16 could be used to identify neonates who will have a poor outcome despite cooling. Stofberg et al.^32^reported similar findings where neonates with Thompson score ≥ 12 were associated with abnormal aEEGs, indicative of HIE. Non-operative deliveries, lack of a doctor at the time of delivery and neonatal chest compressions were also associated with abnormal aEEGs. Doreswamy and Ramakrishnegowda^28^ conducted a study to validate Prediction of Encephalopathy in Perinatal Asphyxia (PEPA) score by Holdout method where Thompson score between 3 and 5-h of life was used to determine post-test probability of developing encephalopathy. The authors found that PEPA score had a higher sensitivity than National Institute of Child Health and Human Development (NICHD) criteria for prediction of HIE in asphyxiated neonates.

From these studies, a list of 32 factors that might be associated with an abnormal Thompson score was compiled (Appendix B). Combining these with the eight predictors identified from clinician consultation and Neotree data, in total 40 potential predictors were identified. 18 of these predictors were either already included as part of the Thompson score or were not currently collected as part of routine data capture via Neotree, leaving 22 predictors to consider (Appendix B).

## Modelling

### Study population

In total 9,445 newborns were admitted during the study period of October 2020 to December 2022, and 6,109 term neonates (gestation age ≥ 37 weeks) met the inclusion criteria. Neonatal mortality rate among term neonates with a recorded outcome was 112 per 1000 admissions (95% CI = (103, 121)) and among those with Thomson score higher than 10, it was 589 per 1000 admissions (95% CI = (497, 694)).

The Thompson score outcome was missing in 55 (0.9%), of which 3.63% died. These were excluded given the small proportion and a lower case fatality rate than TS > 10 group, resulting in a total sample size of 6,054 neonates. Of the 20 candidate predictors, nine had missing values. For foetal heart rate (0.11%), maternal age (0.33%), and temperature at admission (0.69%) they were imputed using median values of the whole sample. Birthweight (0.26%) was imputed as medians conditional on the gestational age. For categorical predictors, namely meconium-stained liquor passed (9.78%), maternal HIV infection (10.3%), meconium-stained umbilicus (11.1%), place of birth (11.7%), and duration of labour (16.09%) a “missing” category was included in the models (Table 2).

**Table 2:** Demographic characteristics of the study sample (*n* = 6054).

Predictor	Levels	Distribution of variables			Univariate logistic regression	
		Total neonates (*n* = 6054)	TS ≤ 10 (*n* = 5808)	TS > 10 (*n* = 246)	Unadjusted odds ratio (95% CI)	*p*-value
Gender *n* (%)	Female	2557 (42.2)	2463 (96.32)	94 (3.68)	Reference	-
	Male	3488 (57.6)	3336 (95.64)	152 (4.36)	1.19 (0.92- 1.557)	0.19
	Unsure	9 (0.15)	9	-	-	-
Gestational age (weeks), median [Q1-Q3]	-	39.0 [38.0–40.0]	39.0 [38.0–40.0]	39.0 [38.0–40.0]	1.0 (0.918–1.088)	0.998
Heart rate median [Q1-Q3]	-	138.0 [127.0–146.0]	138.0 [128.0–146.0]	127.0 [112.0–143.0]	0.97 (0.963- 0.975)	< 0.001*
	Missing *n* (%)	7 (0.1%)	6 (85.7%)	1 (14.3%)	-	-
Birthweight (grams) median [Q1-Q3]	-	3000.0 [2600.0–3400.0]	3000.0 [2600.0–3400.0]	3025.0 [2750.0–3382.0]	1.00 (0.999- 1.0003)	0.43
	Missing *n* (%)	16 (0.26)	15 (93.8%)	1 (6.2)	-	-
Maternal age (years) median [Q1-Q3]	-	[21-31]			0.98 (0.957- 0.997)	0.025*
	Missing *n* (%)	20 (0.33)	20 (0.33)	-		
Temperature (°C) *n* (%)	Normal (36.5–37.5)	4517 (74.61)	4,380 (96.97)	137 (3.03)	Reference	-
	Hypothermia (< 36.5)	1283 (21.19)	1,177 (91.74)	106 (8.26)	2.88 (2.21- 3.74)	< 0.001*
	Hyperthermia (> 37.5)	254 (4.2)	251 (98.82)	3 (1.18)	0.38 (0.09- 1.02)	0.1
Mode delivery *n* (%)	C-section	1552 (25.6)	1500 (96.65)	52 (3.35)	Reference	-
	Other	4502 (74.4)	4308 (95.69)	194 (4.31)	1.3 (0.96–1.79)	0.1
Trauma (in the form of head swelling) *n* (%)	Normal	5316 (87.8)	5162 (97.1)	154 (2.9)	Reference	-
	Swelling	738 (12.2)	646 (87.53)	92 (12.47)	4.77 (3.63- 6.25)	< 0.001*
Meconium-stained liquor passed *n (*%)	No	2647 (43.72)	2537 (95.84)	110 (4.16)	Reference	-
	Yes	2815 (46.5)	2694 (95.7)	121 (4.3)	1.036 (0.8- 1.35)	0.793
	Missing	592 (9.78)	577 (97.47)	15 (2.53)	0.6 (0.33–1.003)	0.067
Maternal HIV infection *n (*%)	Reactive	529 (8.74)	509 (96.22)	20 (3.78)	0.72 (0.40- 1.27)	0.157
	Non-reactive	4904 (81.0)	4710 (96.04)	194 (3.96)	0.76 (0.52- 1.13)	0.266
	Missing	621 (10.3)	589 (94.85)	32 (5.15)	Reference	-
Antenatal care visits *n (*%)	None	643 (10.6)	612 (95.18)	31 (4.82)	Reference	-
	1 to 3	3616 (59.7)	3475 (96.1)	141 (3.9)	0.801 (0.546- 1.213)	0.275
	≥ 4	1795 (29.6)	1721 (95.88)	74 (4.12)	0.849 (0.558- 1.321)	0.455
Parity *n (*%)	Primiparous	958 (15.8)	914 (95.41)	44 (4.59)	Reference	-
	Multiparous	5096 (84.2)	4894 (96.04)	202 (3.96)	0.857 (0.62- 1.21)	0.366
Pregnancy condition present *n (*%)	Hypertension or pre-eclampsia	805 (13.3)	771 (95.78)	34 (4.22)	1.169 (0.79- 1.68)	0.417
	Other	656 (10.8)	611 (93.14)	45 (6.86)	1.95 (1.38- 2.72)	< 0.001*
	None	4593 (75.9)	4426 (96.36)	167 (3.64)	Reference	-
Risk factor of sepsis *n (*%)	Premature rupture of membrane	445 (7.35)	426 (95.73)	19 (4.27)	1.2 (0.72–1.9)	0.455
	Offensive liquor	285 (4.71)	261 (91.58)	24 (8.42)	2.48 (1.55–3.79)	< 0.001*
	Other	347 (5.73)	322 (92.8)	25 (7.2)	2.09 (1.33–3.17)	0.001*
	None	4977 (82.2)	4799 (96.42)	178 (3.58)	Reference	-
Duration of labour (in hours) *n (*%)	< 14	4010 (66.2)	3847 (95.94)	163 (4.06)	Reference	-
	14–19	710 (11.7)	671 (94.51)	39 (5.49)	1.37 (0.95–1.94)	0.084
	20 +	360 (5.95)	340 (94.44)	20 (5.56)	1.39 (0.84–2.18)	0.18
	Missing	974 (16.1)	950 (97.54)	24 (2.46)	0.596 (0.38–0.9)	0.02*
	Unknown	191 (3.15)	187 (97.91)	4 (2.09)	10.41 (2.64–36.74)	< 0.001*
Meconium-stained umbilicus *n (*%)	Meconium	222 (3.67)	199 (89.64)	23 (10.36)	3.02 (1.87–4.67)	< 0.001*
	Other	5158 (85.2)	4968 (96.32)	190 (3.68)	Reference	-
	Missing	674 (11.1)	641 (95.1)	33 (4.9)	1.35 (0.91–1.94)	0.124
Crying after birth *n (*%)	Yes	4017 (66.4)	4003 (99.65)	14 (0.35)	0.072 (0.03- 0.19)	< 0.001*
	No	1885 (31.1)	1660 (88.06)	225 (11.94)	2.81 (1.4–6.69)	0.009*
	Unknown	152 (2.51)	145 (95.39)	7 (4.61)	Reference	-
Reason for caesarean section *n (*%)	Foetal distress	271 (4.48)	257 (94.8)	14 (5.2)	1.21 (0.66- 2.03)	0.508
	Other reason for C-section	1291 (21.3)	1253 (97.1)	38 (2.9)	0.67 (0.47–0.95)	0.027*
	Other delivery	4492 (74.2)	4298 (95.7)	194 (4.3)	Reference	-
Problems during labour *n (*%)	Significant vaginal bleeding/eclampsia	388 (6.41)	374 (96.4)	14 (3.6)	0.88 (0.49–1.47)	0.653
	Other problems	340 (5.62)	325 (95.6)	14 (4.4)	1.09 (0.61–1.79)	0.761
	None	5326 (88.0)	5109 (95.9)	217 (4.1)	Reference	-
Place of birth *n (*%)	SMCH	4321 (71.4)	4144 (95.9)	177 (4.1)	Reference	-
	Other hospital in Harare	638 (10.5)	613 (96.1)	25 (3.9)	0.96 (0.61–1.44)	0.832
	Other place	389 (6.43)	386 (99.2)	3 (0.8)	0.18 (0.05–0.48)	0.004*
	Missing	706 (11.7)	665 (94.2)	41 (5.8)	1.44 (1.01–2.03)	0.04*
Neonatal outcome *n (*%)	Neonatal death	616 (10.2)	471 (76.5)	145 (23.5)	22.46 (16.61- 30.66)	< 0.001*
	Discharged/other	4880 (80.6)	4814 (98.6)	66 (1.4)	Reference	-
	Missing	558 (9.22)	523 (93.7)	35 (6.3)	4.88 (3.18- 7.38)	< 0.001*

Of these 6,054 neonates, 57.6% were male and 70.7% were born via spontaneous vaginal delivery. 558 (9.22%) neonates did not have any outcomes recorded. Among the rest 5,496 neonates, 88.8% survived to discharge. Mean maternal age was 25 years (SD 7). Mean Thompson score was 1.996, Median Thompson score was zero (IQR 0–2). There were 246 (4.06%) neonates with a Thompson score of greater than 10 equating to a rate of 40 per 1000 admitted neonates. 511 (8.4%) of neonates had a Thompson score (TS) of greater than 7.

For the ‘Total neonates’ column in Table 2, counts and percentages are shown out of 6,054. For the ‘TS ≤ 10’ and ‘TS > 10’ columns, within the group distribution of counts and percentages are shown. For example, in the ‘Gender’ variable, 2557 out of 6054 (42.2%) of total newborns were female. Amongst these 2557 newborns with a Thompson score, 2463 (96.32%) had TS ≤ 10 and 94 (3.68%) had TS > 10. Those with TS > 10 had 22 times higher odds of neonatal death compared to those with TS ≤ 10.

### Logistic regression analysis and predictive modelling

Univariable logistic regression models were fitted to test the research hypothesis regarding the relationship between the selected candidate predictors and Thompson score greater than 10. The Apgar score and respiratory distress were not included in the model despite their strong association with the Thompson score due to collinearity caused by reverse causality. For example, respiratory distress is used to measure Thompson score, and the Apgar score includes assessing tone, which is also used in measuring the Thomspon score. In total 11 out of 20 of the candidate predictors were significantly associated (Table 2).

Although ‘Other conditions’ for pregnancy conditions variable showed significant association (*p*-value < 0.001), it was excluded from multiple logistic regression modelling due to inclusion of multiple and ‘unknown’ pregnancy conditions. Thus, it may not be effective in identifying which of those pregnancy conditions is most predictive of an abnormal Thompson score. Similarly, ‘Other place’ in the place of birth variable was significantly associated (*p* = 0.004) but has a very small number of cases (*n* = 3) and missing place of birth was also significant (*p* = 0.04). Due to uncertainty in defining the location, this variable was excluded from the final model. For duration of labour, only the ‘missing’ category was significant (*p* = 0.02) and was excluded for the same reason.

The remaining eight candidate predictors included in multiple logistic regression analysis were: 1. neonatal heart rate (beats/min); 2. temperature at admission; 3. head shape; 4. reason for caesarean section (C-section); 5. maternal age (years); 6. crying immediately after birth; 7. meconium-stained umbilicus and 8. risk factors of sepsis.

Multivariable logistic regression models were fitted for the final dataset of 6,054 neonates to test the null hypothesis that there was no relationship between these eight predictor variables selected from the univariable analysis and a Thompson score > 10. Table 3 shows the regression coefficients and adjusted odds ratios for this model.

**Table 3:** Regression coefficients and odds ratios for the final model (*n* = 6,054).

Categories of risk factors	Predictor	Estimate	Standard error	Adjusted odds ratio	95% CI	*p*-value
	Intercept	− 0.012	0.654	-	-	*-*
Maternal risk factors	Maternal age (years)	0.001	0.011	1.001	0.979—1.024	0.899
	Offensive liquor (maternal risk factor of sepsis)	0.452	0.261	1.572	0.924—2.58	0.083
	Premature rupture of membrane (maternal risk factor of sepsis)	0.109	0.266	1.116	0.644—1.838	0.681
	Other risk factors of sepsis	0.68	0.263	1.975	1.158—3.256	0.01
Neonatal risk factors	Heart rate	− 0.027	0.003	0.974	0.967—0.98	< 0.001
	Temperature < 36.5 °C	0.804	0.153	2.236	1.652—3.015	< 0.001
	Temperature > 37 °C	− 0.139	0.616	0.87	0.206—2.501	0.822
	Trauma (head swelling)	0.785	0.154	2.193	1.617—2.96	< 0.001
	Meconium-stained umbilicus	0.584	0.272	1.793	1.029–3.003	0.032
	Missing data on meconium-stained umbilicus	− 0.208	0.22	0.813	0.521—1.234	0.345
	Cried at birth	− 2.534	0.48	0.079	0.032—0.216	< 0.001
	Did not cry at birth	0.947	0.405	2.578	1.25—6.26	0.019
Intrapartum risk factors	Foetal distress as caesarean reason	− 0.24	0.316	0.787	0.407—1.415	0.448
	Other reason for caesarean	− 0.037	0.201	0.964	0.641—1.416	0.856

Maternal risk factors of sepsis other than offensive liquor and premature rupture of membrane and neonatal risk factors, such as low neonatal heart rate on admission (< 100 bpm), hypothermia, trauma in the form of head swelling, meconium-stained umbilicus and not crying at birth were associated with higher odds of Thompson score > 10.

## Discussion

To our knowledge, this is the first large-sample prospective study in a low-resource clinical setting exploring associations between abnormal Thompson score and thus risk of NE on admission of term infants (gestation age > 37). NE is a clinical diagnosis in low-resource settings where investigations such as EEG, cranial imaging and umbilical cord gases are typically unavailable. Thompson score is a key scoring system used to identify at-risk neonates in clinical settings. However, it may be unclear which neonates should be assessed for clinical signs of NE and thus necessitate the measurement of Thompson score. This study aimed to identify key risk factors of neonates with abnormal Thompson score (> 10) based on a literature review of studies conducted in similar settings and routine data collected using Neotree from 6,054 neonates admitted at a tertiary-level hospital in Zimbabwe. The prevalence of abnormal Thompson score (as a proxy of diagnosis of NE) in this population of term neonates was 40 per 1000 with 58.9% (145 out of 246) of these neonates not surviving. It is not possible to compare this with prevalence estimates per 1000 live births due to lack of data. These estimates are lower than a recent study in Nigeria reporting prevalence of 71 per 1000 admissions with fatality rate of 25.3%^35^. In this study, majority of the infants were born outside, and place of birth was associated with higher fatality rate. Although our study sample has a smaller proportion of outborn neonates, univariate logistic regression showed similar significant results for that category.

Initially, 40 predictors were identified based on literature review and consultations with clinicians in Zimbabwe. Of these, 22 were selected to be the most important. Six risk factors were identified using logistic regression, the presence of which might prompt healthcare providers to undertake a neurological examination and calculate a Thompson score. These are the most predictive covariates for determining a Thompson score of greater than 10: low neonatal heart rate on admission (< 100 bpm), hypothermia, trauma in the form of head swelling, maternal risk factors of sepsis other than offensive liquor and premature rupture of membrane, meconium-stained umbilicus and not crying at birth were associated with higher odds of Thompson score > 10.

In this study sample, 90.65% (*n*= 223) of neonates with an abnormal Thompson score had a low Apgar score of < 7 at 5-min, which is in line with the studies from scoping review where all studies used low Apgar score as an inclusion criterion. Similar studies found significant association of NE or serious neurologic dysfunction with Apgar score at 5-min^31^^,^^36^. This is plausible since several criteria used to determine Apgar score, such as muscle tone and respiration are also used to derive Thompson score. Similarly, low neonatal heart rate is associated with low Apgar score and was also found to be associated with high Thompson score in our model. Low neonatal heart rate on admission suggests that neonates who may be in extremis are being transferred to the neonatal unit reflecting severity of insult around the time of birth.

Although maternal pre-eclampsia was one of the prenatal diagnoses reported by Biselele et al.^26^ and Biselele et al.^16,^it was not significantly associated in this study population. Some studies also reported inappropriate or absence of antenatal care as a maternal risk factor while this was not strongly associated in this study population^31^^,^^25^. Additionally, this study identified that maternal risk factor of sepsis, other than premature rupture of membrane and offensive liquor can increase the odds of Thompson score greater than 10.

A key strength of this study is that the a priori variables used in predictive modelling were assembled using a combination of evidence from the literature, routine health records and clinically driven reasoning. In this way, the study is grounded in the lived experiences of clinicians, data collection systems and healthcare providers in low-resource settings who often have limited data and resources needed save the lives of infants. Compared to previous studies that modelled risk factors of NE^31^, this paper also relied on a larger sample, thus has greater statistical power.

## Limitations

A limitation in this study is the small number of missing values in most variables and due to evidence against missingness at random, we dealt with this by adding a “missing” category thus increasing the model’s degrees of freedom. Some confidence intervals were wide, possibly due to the number of study participants, decreasing statistical power. The study did not include some variables like intra-uterine growth restriction (IUGR) in the model due to non-availability. Also, IUGR contributes to high Thompson score values, low Apgar score and low heart rate, and could cause omitted variable bias. Birth weight was used as a proxy measure of IUGR, although significant associations were not found. In addition, while some of the variables had significant effects, such as ‘Other risk factors of sepsis’ for admission, it may be complicated to apply within the Neotree system given this group can have a variety of admission reasons.

## Conclusions

This study aimed to address the lack of well-defined clinical triggers for suspecting NE and thus performing the Thompson score in neonates in low-resource settings. With the objective to construct an empirically driven and parsimonious model for identifying Thompson score > 10 in term neonates, this study identified six risk factors that are most predictive. These include five neonatal factors – low neonatal heart rate at admission, hypothermia at admission, trauma in the form of head swelling, baby not crying at birth, meconium-stained umbilicus; and one intrapartum factor– maternal risk factors of sepsis other than premature rupture of membrane and offensive liquor.

The results imply that integration of these risk factors into clinical decision support systems such as Neotree would enable healthcare professionals to identify neonates to screen for NE using the Thompson score. However, before operationalisation into the application, further work is needed to evaluate the model’s performance through sensitivity and specificity analysis. This evaluation should ensure a large enough sample of Thompson scores to allow for the dichotomisation of predictors such as heart rate. Finally, future work could also focus on using more novel predictive model building techniques that use artificial intelligence and machine learning.

## Supplementary Information

Supplementary Information 1.

Supplementary Information 2.

## Data Availability

The datasets generated and/or analysed during the current study are not publicly available as the data belongs to the Zimbabwean Ministry of Health but anonymised data are available from the corresponding author on reasonable request.

## Abbreviations

NE: Neonatal encephalopathy
HIE: Hypoxic Ischaemic Encephalopathy
EEG: Electroencephalogram
MRI: Magnetic resonance imaging
TS: Thompson score
aEEG: Amplitude-integrated electroencephalogram
NICHD: National Institute of Child Health and Human Development
