# Supplementary material for: A scoping review and modelling of predictors of an abnormal Thompson score in term neonates in low-resource settings
Source: Sci Rep. 2025 Apr 10;15:12217. doi: 10.1038/s41598-025-96566-7 (PMC11986035; doi:10.1038/s41598-025-96566-7)
Supplement: Supplementary file 1 — Supplementary Information 1. [file 41598_2025_96566_MOESM1_ESM.docx]

**Search strings:**

**PubMed string combining all three scores**

(newborn OR neonate) AND ("Thompson scor*" OR "Hypoxic ischaemic encephalopathy scor*" OR "Sarnat Scor*" OR “Sarnat”) AND ("Hypoxia-Ischemia, Brain"[Mesh] OR "hypoxic ischaemic encephalopathy" OR "neonatal encephalopathy" OR "birth asphyxia" OR "asphyxia" OR “perinatal asphyxia”) AND ("Developing Countries"[Mesh] OR LMIC OR "low resource" OR afghanistan[MeSH] OR albania[MeSH] OR algeria[MeSH] OR “american samoa”[MeSH] OR angola[MeSH] OR “antigua and barbuda”[MeSH] OR argentina[MeSH] OR armenia[MeSH] OR aruba[MeSH] OR azerbaijan[MeSH] OR bahrain[MeSH] OR bangladesh[MeSH] OR barbados[MeSH] OR “republic of belarus”[MeSH] OR belize[MeSH] OR benin[MeSH] OR bhutan[MeSH] OR bolivia[MeSH] OR “bosnia and herzegovina”[MeSH] OR botswana[MeSH] OR brazil[MeSH] OR bulgaria[MeSH] OR “burkina faso”[MeSH] OR burundi[MeSH] OR cabo verde[MeSH] OR cambodia[MeSH] OR cameroon[MeSH] OR “central african republic”[MeSH] OR chad[MeSH] OR chile[MeSH] OR china[MeSH] OR colombia[MeSH] OR comoros[MeSH] OR “democratic republic of the congo”[MeSH] OR congo[MeSH] OR “costa rica”[MeSH] OR “cote d’ivoire”[MeSH] OR croatia[MeSH] OR cuba[MeSH] OR cyprus[MeSH] OR “czech republic”[MeSH] OR djibouti[MeSH] OR dominica[MeSH] OR “dominican republic”[MeSH] OR ecuador[MeSH] OR egypt[MeSH] OR “el salvador”[MeSH] OR “equatorial guinea”[MeSH] OR eritrea[MeSH] OR estonia[MeSH] OR swaziland[MeSH] OR ethiopia[MeSH] OR fiji[MeSH] OR gabon[MeSH] OR gambia[MeSH] OR georgia (republic) [MeSH] OR ghana[MeSH] OR gibraltar[MeSH] OR greece[MeSH] OR grenada[MeSH] OR guam[MeSH] OR guatemala[MeSH] OR guinea[MeSH] OR “guinea bissau”[MeSH] OR guyana[MeSH] OR haiti[MeSH] OR honduras[MeSH] OR hungary[MeSH] OR india[MeSH] OR indonesia[MeSH] OR iran[MeSH] OR iraq[MeSH] OR jamaica[MeSH] OR jordan[MeSH] OR kazakhstan[MeSH] OR kenya[MeSH] OR “democratic people’s republic of korea”[MeSH] OR “republic of korea”[MeSH] OR kosovo[MeSH] OR kyrgyzstan[MeSH] OR laos[MeSH] OR latvia[MeSH] OR lebanon[MeSH] OR lesotho[MeSH] OR liberia[MeSH] OR libya[MeSH] OR lithuania[MeSH] OR macau[MeSH] OR “republic of north macedonia”[MeSH] OR madagascar[MeSH] OR malawi[MeSH] OR malaysia[MeSH] OR “indian ocean islands”[MeSH] OR mali[MeSH] OR malta[MeSH] OR micronesia[MeSH] OR palau[MeSH] OR mauritania[MeSH] OR mauritius[MeSH] OR mexico[MeSH] OR moldova[MeSH] OR mongolia[MeSH] OR montenegro[MeSH] OR morocco[MeSH] OR mozambique[MeSH] OR myanmar[MeSH] OR namibia[MeSH] OR nepal[MeSH] OR “netherlands antilles”[MeSH] OR nicaragua[MeSH] OR niger[MeSH] OR nigeria[MeSH] OR oman[MeSH] OR pakistan[MeSH] OR panama[MeSH] OR “papua new guinea”[MeSH] OR paraguay[MeSH] OR peru[MeSH] OR philippines[MeSH] OR poland[MeSH] OR portugal[MeSH] OR “puerto rico”[MeSH] OR romania[MeSH] OR russia[MeSH] OR rwanda[MeSH] OR samoa[MeSH] OR “sao tome and principe”[MeSH] OR “saudi arabia”[MeSH] OR senegal[MeSH] OR serbia[MeSH] OR seychelles[MeSH] OR “sierra leone”[MeSH] OR slovakia[MeSH] OR slovenia[MeSH] OR melanesia[MeSH] OR somalia[MeSH] OR “south africa”[MeSH] OR “south sudan”[MeSH] OR “sri lanka”[MeSH] OR “saint kitts and nevis”[MeSH] OR “saint lucia”[MeSH] OR “saint vincent and the grenadines”[MeSH] OR sudan[MeSH] OR suriname[MeSH] OR syria[MeSH] OR tajikistan[MeSH] OR tanzania[MeSH] OR thailand[MeSH] OR “timor leste”[MeSH] OR togo[MeSH] OR tonga[MeSH] OR “trinidad and tobago”[MeSH] OR tunisia[MeSH] OR turkey[MeSH] OR turkmenistan[MeSH] OR uganda[MeSH] OR ukraine[MeSH] OR uruguay[MeSH] OR uzbekistan[MeSH] OR vanuatu[MeSH] OR venezuela[MeSH] OR vietnam[MeSH] OR “middle east”[MeSH] OR yemen[MeSH] OR yugoslavia[MeSH] OR zambia[MeSH] OR zimbabwe[MeSH] OR “africa south of the sahara”[MeSH] OR “africa, central”[MeSH] OR “africa, northern”[MeSH] OR “africa, southern”[MeSH] OR “africa, eastern”[MeSH] OR “africa, western”[MeSH] OR “west indies”[MeSH] OR “indian ocean islands”[MeSH] OR “caribbean region”[MeSH] OR “central america”[MeSH] OR “latin america”[MeSH] OR “south america”[MeSH] OR “asia, central”[MeSH] OR “asia, northern”[MeSH] OR “asia, southeastern”[MeSH] OR “asia, western”[MeSH] OR “europe, eastern”[MeSH])

**SCOPUS string combining all three scores**

TITLE-ABS-KEY ( ( newborn  OR  neonate )  AND  ( "Thompson scor*"  OR  "Sarnat scor*"  OR  "Sarnat"  OR  "Hypoxic ischaemic encephalopathy scor*" )  AND  ( "hypoxic ischaemic encephalopathy"  OR  "neonatal encephalopathy"  OR  "birth asphyxia"  OR  "perinatal asphyxia"  OR  "asphyxia" ) )  AND  ( "developing countries"  OR  "low income countries"  OR  "low resource"  OR  "low resource settings"  OR  "africa south of the sahara"  OR  "central africa"  OR  "northern africa"  OR  "southern africa"  OR  "eastern africa"  OR  "western africa"  OR  "west indies"  OR  "indian ocean islands"  OR  "caribbean region"  OR  "central america"  OR  "latin america"  OR  "south america"  OR  "central asia"  OR  "northern asia"  OR  "southeastern asia"  OR  "western asia"  OR  "eastern europe" )

**Web of Science**

TS=((newborn OR neonate) AND ("Thompson scor*" OR "Hypoxic ischaemic encephalopathy scor*" OR "Sarnat Scor*" OR “Sarnat”) AND ("Hypoxia-Ischemia, Brain" OR "hypoxic ischaemic encephalopathy" OR "neonatal encephalopathy" OR "birth asphyxia" OR "asphyxia" OR “perinatal asphyxia”) AND ("developing countries" OR "low resource" OR "low income countries" OR "low resource settings" OR afghanistan OR albania OR algeria OR “american samoa” OR angola OR “antigua and barbuda” OR argentina OR armenia OR aruba OR azerbaijan OR bahrain OR bangladesh OR barbados OR “republic of belarus” OR belize OR benin OR bhutan OR bolivia OR “bosnia and herzegovina” OR botswana OR brazil OR bulgaria OR “burkina faso” OR burundi OR cabo verde OR cambodia OR cameroon OR “central african republic” OR chad OR chile OR china OR colombia OR comoros OR “democratic republic of the congo” OR congo OR “costa rica” OR “cote d’ivoire” OR croatia OR cuba OR cyprus OR “czech republic” OR djibouti OR dominica OR “dominican republic” OR ecuador OR egypt OR “el salvador” OR “equatorial guinea” OR eritrea OR estonia OR swaziland OR ethiopia OR fiji OR gabon OR gambia OR georgia (republic)  OR ghana OR gibraltar OR greece OR grenada OR guam OR guatemala OR guinea OR “guinea bissau” OR guyana OR haiti OR honduras OR hungary OR india OR indonesia OR iran OR iraq OR jamaica OR jordan OR kazakhstan OR kenya OR “democratic people’s republic of korea” OR “republic of korea” OR kosovo OR kyrgyzstan OR laos OR latvia OR lebanon OR lesotho OR liberia OR libya OR lithuania OR macau OR “republic of north macedonia” OR madagascar OR malawi OR malaysia OR “indian ocean islands” OR mali OR malta OR micronesia OR palau OR mauritania OR mauritius OR mexico OR moldova OR mongolia OR montenegro OR morocco OR mozambique OR myanmar OR namibia OR nepal OR “netherlands antilles” OR nicaragua OR niger OR nigeria OR oman OR pakistan OR panama OR “papua new guinea” OR paraguay OR peru OR philippines OR poland OR portugal OR “puerto rico” OR romania OR russia OR rwanda OR samoa OR “sao tome and principe” OR “saudi arabia” OR senegal OR serbia OR seychelles OR “sierra leone” OR slovakia OR slovenia OR melanesia OR somalia OR “south africa” OR “south sudan” OR “sri lanka” OR “saint kitts and nevis” OR “saint lucia” OR “saint vincent and the grenadines” OR sudan OR suriname OR syria OR tajikistan OR tanzania OR thailand OR “timor leste” OR togo OR tonga OR “trinidad and tobago” OR tunisia OR turkey OR turkmenistan OR uganda OR ukraine OR uruguay OR uzbekistan OR vanuatu OR venezuela OR vietnam OR “middle east” OR yemen OR yugoslavia OR zambia OR zimbabwe OR “africa south of the sahara” OR “central africa” OR “northern africa” OR “southern africa” OR “eastern africa” OR “western africa” OR “west indies” OR “indian ocean islands” OR “caribbean region” OR “central america” OR “latin america” OR “south america” OR “central asia” OR “northern asia” OR “southeastern asia” OR “western asia” OR “eastern europe”))
