# Supplementary material for: A scoping review and modelling of predictors of an abnormal Thompson score in term neonates in low-resource settings
Source: Sci Rep. 2025 Apr 10;15:12217. doi: 10.1038/s41598-025-96566-7 (PMC11986035; doi:10.1038/s41598-025-96566-7)
Supplement: Supplementary file 2 — Supplementary Information 2. [file 41598_2025_96566_MOESM2_ESM.docx]

**Appendix A:**  Determination of a priori variables

|  | **Variable** | **Strong evidence base from literature** | **Based on NeoTree Delphi study** | **Clinically driven but scant evidence** | **Final decision** | **Summary of comments from neonatal experts** |
| --- | --- | --- | --- | --- | --- | --- |
| **Maternal triggers** | | | | | | |
| 1 | Mode of delivery: c-section | **✓** | **✓** |  | Include | Would be important to distinguish between elective or emergency c-section |
| 2 | Maternal age | **✓** |  |  | Include |  |
| 3 | No. of antenatal visits | **✓** |  |  | Include |  |
| 4 | Gravidity and parity | **✓** |  |  | Include | Only parity is recorded in the NeoTree app |
| 5 | Pregnancy-induced hypertension or Pre-eclampsia | **✓** |  |  | Include |  |
| 6 | Meconium-stained liquor | **✓** |  |  | Include | Would be important to distinguish between thick or thin liquor |
| 7 | Post-term pregnancy | **✓** |  |  | Include |  |
| 8 | PROM (Premature rupture of membranes) | **✓** |  |  | Include |  |
| 9 | Prolonged labour (first/second stage) | **✓** |  |  | Include |  |
| 10 | Maternal haemorrhage | **✓** |  |  | Include |  |
|  | **Neonatal Triggers** | | | | | |
| 11 | Gestational age (term) | **✓** |  |  | Include |  |
| 12 | Low birth weight | **✓** |  |  | Include |  |
| 13 | Meconium-stained infant | **✓** |  |  | Include |  |
| 14 | Abnormal foetal heart rate | **✓** |  |  | Include |  |
| 15 | Delayed crying | **✓** |  |  | Include |  |
| 16 | Gender | **✓** |  |  | Include |  |
| 17 | Infection – HIV |  |  | **✓** | Include | Include as these mothers tend to have poorer antenatal care |
| 18 | Trauma |  |  | **✓** | Include | Could be in the form of head swelling |
| 19 | Foul smelling liquor |  |  | **✓** | Include |  |
| 20 | Hypothermia |  |  | **✓** | Include |  |
|  | **Intrapartum triggers** | | | | | |
| 21 | Foetal distress | **✓** | **✓** |  | Include |  |
| 22 | Birthplace: (e.g., home birth) |  |  | **✓** | Include | Include as there can be uncertainty on duration of labour, foetal monitoring. Home birth can be unassisted and cause trauma and thus, baby could require resuscitation. |
|  | Other triggers (factors already part of Thompson Score (*) or that are not in the app (†)) | | | | | |
| 23 | Posturing movement disorder | **✓** |  |  | Exclude | * |
| 24 | Activity: Alert, lethargic, convulsions | **✓** | **✓** |  | Exclude | * |
| 25 | Activity: coma |  | **✓** |  | Exclude | * |
| 26 | Impaired sucking, swallowing | **✓** |  |  | Exclude | * |
| 27 | Impaired feeding | **✓** |  |  | Exclude | * |
| 28 | Respiratory distress |  | **✓** |  | Exclude | * |
| 29 | Resuscitation lasting 10 minutes or longer | **✓** | **✓** |  | Exclude | * |
| 30 | Low 5-minute Apgar score (<7) | **✓** | **✓** |  | Exclude | * |
| 31 | Tone: High |  | **✓** |  | Exclude | * |
| 32 | Urogenital infection | **✓** |  |  | Exclude | † |
| 33 | Intrauterine growth restriction | **✓** |  |  | Exclude | † |
| 34 | Placenta previa | **✓** |  |  | Exclude | † |
| 35 | Maternal trauma | **✓** |  |  | Exclude | † |
| 36 | Maternal cardio-respiratory arrest | **✓** |  |  | Exclude | † |
| 37 | Cord Prolapse | **✓** |  |  | Exclude | † |
| 38 | Uterine rupture | **✓** |  |  | Exclude | † |
| 39 | Shoulder dystocia | **✓** |  |  | Exclude | † |
| 40 | Blood pH levels/base deficit | **✓** |  |  | Exclude | † |
